# Ozone-related acute excess mortality projected to increase in the absence of climate and air quality controls consistent with the Paris Agreement

**DOI:** 10.1016/j.oneear.2024.01.001

**Published:** 2024-02-16

**Authors:** Nina G.G. Domingo, Arlene M. Fiore, Jean-Francois Lamarque, Patrick L. Kinney, Leiwen Jiang, Antonio Gasparrini, Susanne Breitner, Eric Lavigne, Joana Madureira, Pierre Masselot, Susana das Neves Pereira da Silva, Chris Fook Sheng Ng, Jan Kyselý, Yuming Guo, Shilu Tong, Haidong Kan, Aleš Urban, Hans Orru, Marek Maasikmets, Mathilde Pascal, Klea Katsouyanni, Evangelia Samoli, Matteo Scortichini, Massimo Stafoggia, Masahiro Hashizume, Barrak Alahmad, Magali Hurtado Diaz, César De la Cruz Valencia, Noah Scovronick, Rebecca M. Garland, Ho Kim, Whanhee Lee, Aurelio Tobias, Carmen Íñiguez, Bertil Forsberg, Christofer Åström, Martina S. Ragettli, Yue Leon Guo, Shih-Chun Pan, Valentina Colistro, Michelle Bell, Antonella Zanobetti, Joel Schwartz, Alexandra Schneider, Ana M. Vicedo-Cabrera, Kai Chen

**Affiliations:** 1 Department of Environmental Health Sciences, Yale School of Public Health, New Haven, CT 06510, USA; 2 Yale Center on Climate Change and Health, Yale School of Public Health, New Haven, CT 06510, USA; 3 Department of Earth, Atmospheric, and Planetary Sciences, Massachusetts Institute of Technology, Cambridge, MA 02139, USA; 4 Climate and Global Dynamics Laboratory, National Center for Atmospheric Research, Boulder, CO, USA; 5 Department of Environmental Health, School of Public Health, Boston University, Boston, MA 02118, USA; 6 Asian Demographic Research Institute, Shanghai University, Shanghai 200444, China; 7 Population Council, New York, NY 10017, USA; 8 Environment & Health Modelling (EHM) Lab, Department of Public Health Environments and Society, London School of Hygiene & Tropical Medicine, London, UK; 9 IBE-Chair of Epidemiology, Faculty of Medicine, LMU Munich, Munich, Germany; 10 Institute of Epidemiology, Helmholtz Zentrum München – German Research Center for Environmental Health, Neuherberg, Germany; 11 School of Epidemiology & Public Health, Faculty of Medicine, University of Ottawa, Ottawa, ON, Canada; 12 Environmental Health and Science Bureau, Heatlh Canada, Ottawa, ON, Canada; 13 Environmental Health Department of the National Health Institute of Health Dr. Ricardo Jorge, Porto, Portugal; 14 EPIUnit - Instituto de Saúde Pública, Universidade do Porto, Porto, Portugal; 15 Laboratório para a Investigação Integrativa e Translacional em Saúde Populacional (ITR), Porto, Portugal; 16 Department of Public Health Environments and Society, London School of Hygiene & Tropical Medicine, London, UK; 17 Department of Global Health Policy, Graduate School of Medicine, The University of Tokyo, Tokyo, Japan; 18 School of Tropical Medicine and Global Health, Nagasaki University, Nagasaki, Japan; 19 Institute of Atmospheric Physics, Czech Academy of Sciences, Prague, Czech Republic; 20 Faculty of Environmental Sciences, Czech University of Life Sciences, Prague, Czech Republic; 21 Department of Epidemiology and Preventive Medicine, School of Public Health and Preventive Medicine, Monash University, Melbourne, NSW, Australia; 22 Climate, Air Quality Research Unit, School of Public Health and Preventive Medicine, Monash University, Melbourne, NSW, Australia; 23 School of Public Health and Social Work, Queensland University of Technology, Brisbane, QLD, Australia; 24 School of Public Health and Institute of Environment and Human Health, Anhui Medical University, Hefei, China; 25 Shanghai Children’s Medical Centre, Shanghai Jiao-Tong University, Shanghai, China; 26 Department of Environmental Health, School of Public Health, Fudan University, Shanghai, China; 27 Department of Family Medicine and Public Health, University of Tartu, Tartu, Estonia; 28 Estonian Environmental Research Centre, Tallinn, Estonia; 29 Santé Publique France, Department of Environmental Health, French National Public Health Agency, Saint Maurice, France; 30 Department of Hygiene, Epidemiology and Medical Statistics, National and Kapodistrian University of Athens, Athens, Greece; 31 Environmental Research Group, School of Public Health, Imperial College London, London, UK; 32 Department of Epidemiology, Lazio Regional Health Service, Rome, Italy; 33 Department of Environmental Health, Harvard T.H. Chan School of Public Health, Harvard University, Boston, MA, USA; 34 Department of Environmental Health, National Institute of Public Health, Cuernavaca, Morelos, Mexico; 35 Department of Environmental Health, Rollins School of Public Health, Emory University, Atlanta, GA, USA; 36 Department of Geography, Geoinformatics and Meteorology, University of Pretoria, Pretoria, South Africa; 37 Graduate School of Public Health, Seoul National University, Seoul, Republic of Korea; 38 Department of Occupational and Environmental Medicine, College of Medicine, Ewha Womans University, Seoul, Republic of Korea; 39 Institute of Ewha-SCL for Environmental Health (IESEH), Seoul, Republic of Korea; 40 Institute of Environmental Assessment and Water Research (IDAEA), Spanish Council for Scientific Research (CSIC), Barcelona, Spain; 41 Department of Statistics and Computational Research, Universitat de València, València, Spain; 42 Ciberesp, Madrid, Spain; 43 Department of Public Health and Clinical Medicine, Umeå University, Umeå, Sweden; 44 Swiss Tropical and Public Health Institute, Basel, Switzerland; 45 University of Basel, Basel, Switzerland; 46 Environmental and Occupational Medicine, National Taiwan University (NTU) College of Medicine and NTU Hospital, Taipei, Taiwan; 47 National Institute of Environmental Health Science, National Health Research Institutes, Zhunan, Taiwan; 48 The Centre on Climate Change and Planetary Health, London School of Hygiene & Tropical Medicine, London, UK; 49 Department of Quantitative Methods, School of Medicine, University of the Republic, Montevideo, Uruguay; 50 School of the Environment, Yale University, New Haven, CT, USA; 51 Institute of Social and Preventive Medicine, University of Bern, Bern, Switzerland; 52 Oeschger Center for Climate Change Research, University of Bern, Bern, Switzerland

## Abstract

Short-term exposure to ground-level ozone in cities is associated with increased mortality and is expected to worsen with climate and emission changes. However, no study has yet comprehensively assessed future ozone-related acute mortality across diverse geographic areas, various climate scenarios, and using CMIP6 multi-model ensembles, limiting our knowledge on future changes in global ozone-related acute mortality and our ability to design targeted health policies. Here, we combine CMIP6 simulations and epidemiological data from 406 cities in 20 countries or regions. We find that ozone-related deaths in 406 cities will increase by 45 to 6,200 deaths/year between 2010 and 2014 and between 2050 and 2054, with attributable fractions increasing in all climate scenarios (from 0.17% to 0.22% total deaths), except the single scenario consistent with the Paris Climate Agreement (declines from 0.17% to 0.15% total deaths). These findings stress the need for more stringent air quality regulations, as current standards in many countries are inadequate.

## Graphical abstract

**Graphical abstract:**
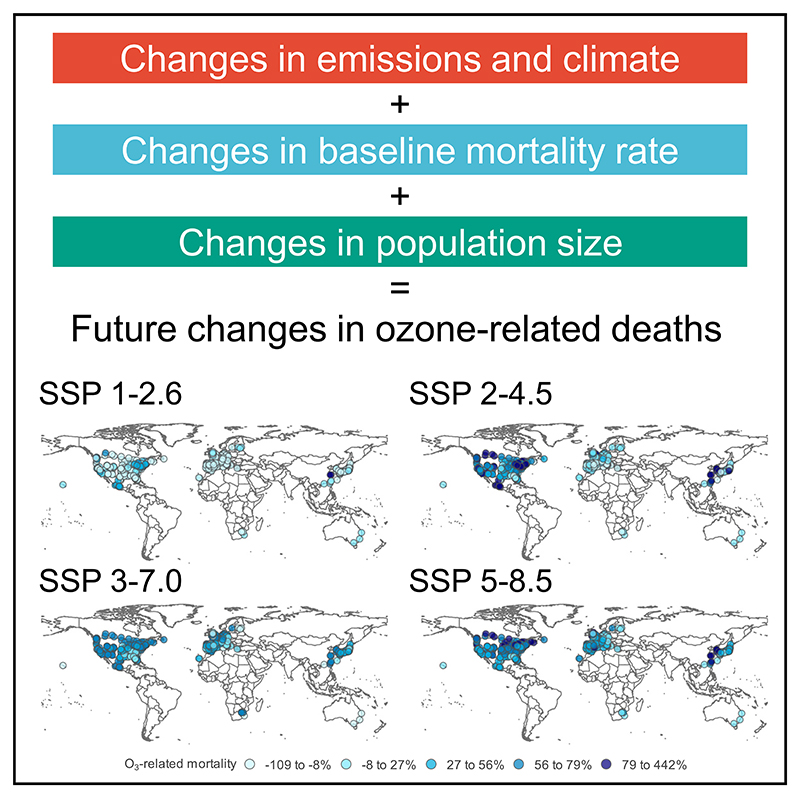

## Introduction

Poor air quality is the largest environmental risk to human health, accounting for 6.7 million deaths of the total 9 million pollution-related deaths in 2019.^1^ Among the different types of air pollutants, ground-level ozone is a highly reactive and oxidative gas that can trigger coughing and shortness of breath, worsen asthma, and cause damage to airways.^2^ Short-term exposure to ozone has been linked to heighted excess mortality due to respiratory and cardiovascular disease^2^ as well as to non-injury and all-cause mortality.^3,4^ A recent multi-city multi-country study found that the ozone-mortality relationship is spatially heterogeneous, with different countries and regions experiencing different health impacts from short-term ozone exposure.^5^

Climate change and changes in emissions of ozone precursors are projected to worsen ground-level ozone concentrations in many areas around the world but with the magnitude of change varying across regions.^6^ The spatial heterogeneity of future changes in ozone production and loss are driven by spatial differences in anthropogenic and natural emissions of ozone precursors as well as spatial differences in meteorological parameters, such as temperature, water vapor, and radiation.^7^ The spatial heterogeneity observed in both future ozone concentrations and ozone-mortality relationships suggest the importance of conducting health impact assessments with broad geographical coverage for designing nuanced policies linked to health promotion and disease prevention.

Previous research has projected varied impacts on ozone-related acute excess mortality, ranging from minor to significant increases in scenarios characterized by high global warming and elevated emissions of ozone precursors. Conversely, in scenarios with low global warming and reduced emissions of ozone precursors, the projected health impacts range from moderate decreases to small increases.^8–10^ However, these studies have generally been limited in geographical coverage, with particular focus on the United States and China, and have only considered a narrow range of future climate and air quality scenarios.^8–10^ No study has yet estimated future changes in ozone-related acute excess mortality across a broad geographical scope and across a broader range of future climate and air quality scenarios. This limits our ability to understand the magnitude of the future health burden and to design effective policies to protect public health.

Here, we assess the near-future (2050–2054) changes in ozone-related short-term excess mortality in 406 cities in 20 countries or regions due to changes in emissions and climate, baseline mortality rates, and population under four shared socioeconomics pathways (SSP) scenarios. The analysis is conducted using simulated maximum daily 8-h average ozone concentrations from five models from the most recent Coupled Model Intercomparison Project Phase 6 (CMIP6), projections of population and baseline mortality rates from the SSP project, and observed ozone concentrations and country- or region-specific ozone-mortality relationships from a recent study by the Multi-Country Multi-City (MCC) Collaborative Research Network. We find that ozone-related acute excess mortality increases across all studied scenarios and is accompanied by rising attributable mortality fractions in all instances except for the scenario that aligns with the objectives of the Paris Climate Agreement. These results underscore the significance of developing urgent and location-specific air pollution and climate mitigations to minimize ozone-related health burden.

## Results

### Results overview

This study estimates the near-future (2050–2054) changes in ozone-related short-term excess mortality by following the causal pathway of ozone concentrations to population exposure (see our methods in the experimental procedures section). In the following subsections, we describe our findings at each stage of the analysis. To concisely present our findings, we aggregate the results obtained from specific sites to the country or region level; however, it is important to note that these aggregations should not be construed as being representative of the entire country or region.

### Present and future ozone concentrations

**Figure 1:**
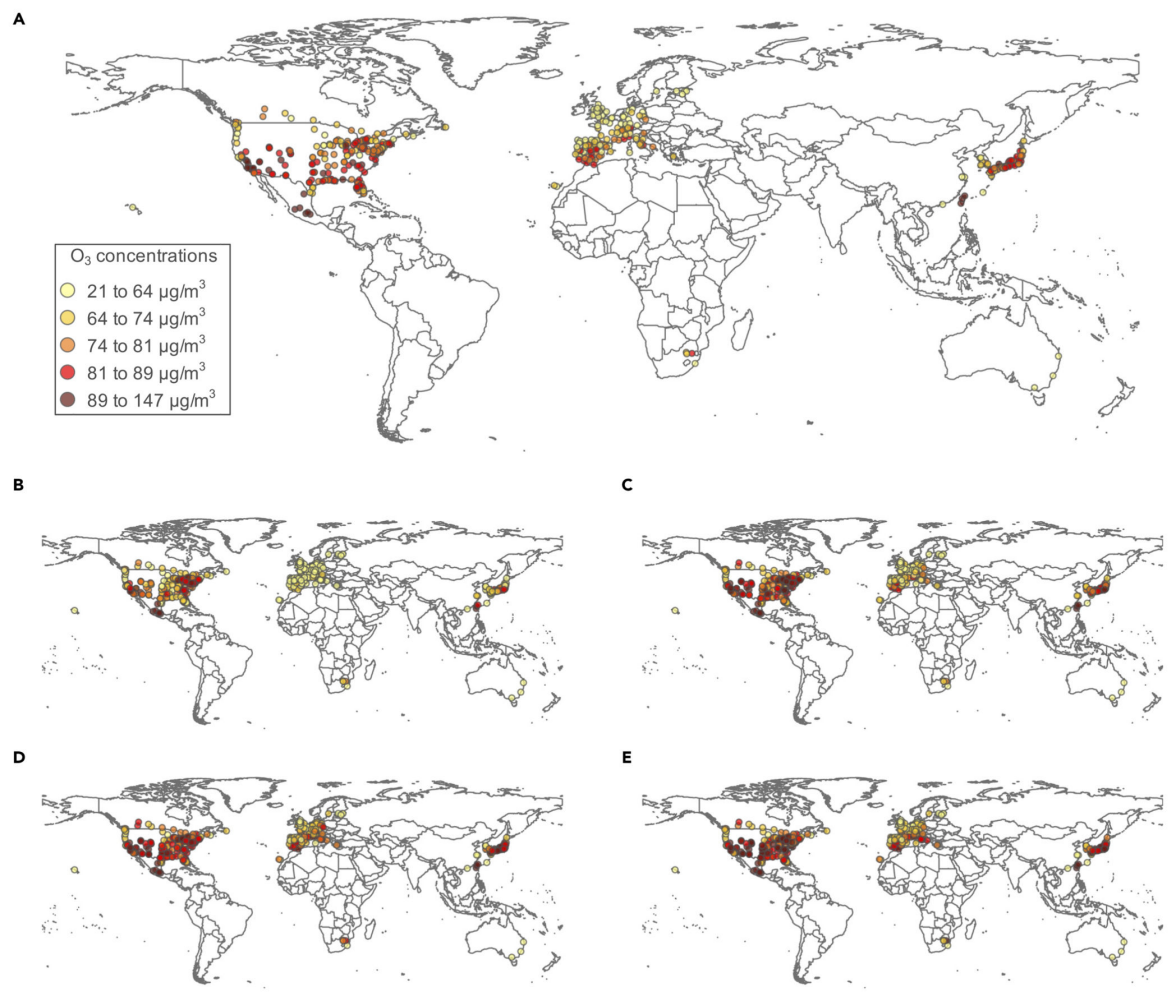
Annual average MDA8 O_3_ concentration at 406 locations in 20 countries or regions in present (2010–2014) and future (2050–2054) time periods in the AerChemMIP models (A) Present O_3_ concentrations in each city, (B) absolute change in O_3_ concentrations under SSP1-2.6, (C) absolute change in O_3_ concentrations under SSP2-4.5, (D) absolute change in O_3_ concentrations under SSP3-7.0, and (E) absolute change in O_3_ concentrations under SSP5-8.5. O_3_ concentration is the maximum daily 8-h average.

Between 2010 and 2014, the annual mean bias-corrected ozone concentration across the 406 studied cities was 73 μg/m^3^. As shown in Figure 1A, annual mean daily maximum 8-h average (MDA8) ozone concentrations varied spatially, with higher concentrations found in Mexico, Taiwan, Japan, the United States, Canada, and Japan, while lower concentrations were found in Australia, Northern Europe, and China. On average, ozone concentrations exceeded the maximum background levels of 70 μg/m^3^,a threshold assumed by past health impact assessments in which concentrations are largely attributed to non-anthropogenic sources,^5^ about 222 days per year in the studied cities.

Under a scenario with strong climate and air pollution controls (SSP1-2.6), ozone concentrations are projected to decrease on average by 8 μg/m^3^ between the present and future periods. In this scenario, ozone concentrations are projected to decrease in nearly all studied countries or regions except in Canada and China. The middle-of-the-road scenario (SSP2-4.5) is linked to a slight overall increase of 1 μg/m^3^ in ozone concentrations, with projected increases in Asia, North America, Czech Republic, Estonia, Germany, and the UK and projected decreases in South Africa and the remaining studied countries or regions in Europe. The regional rivalry scenario (SSP3-7.0), which has weak climate and air pollution controls, is linked to an average 9 μg/m^3^ increase in ozone concentrations across all countries or regions. Similarly, the scenario with weak climate mitigation measures but strong air pollution measures (SSP5-8.5) is linked to an average increase of 4 μg/m^3^ ozone concentrations across all studied countries or regions (Table S1). Though the number of models available for each SSP varies, in general, the UKESM-1-0-LL and MPI-ESM-1-2-HAM models project higher ozone concentrations than the other models in the present period.

A bias-correction technique was performed on simulated ozone concentrations from five global chemistry-climate models using observed concentrations from the MCC network to obtain city-level concentrations for the present (2010–2014) and future (2050–2054) periods. In the present period, simulated annual mean ozone concentrations on average overestimate observed ozone concentrations by about 4 μg/m^3^ (range: −75 to 50 μg/m^3^) (Figure S1). Biases varied spatially, however, with simulated ozone concentrations generally underestimating ozone concentrations in North America and Taiwan (range: 6–41 μg/m^3^ below observed concentrations) and overestimating ozone concentrations in all other studied countries or regions (range: 4–32 μg/m^3^ above observed concentrations) (Figure S4).

The spatial distribution and sign of changes in ozone concentrations are generally consistent across models for a given SSP, though there are a few key differences. For instance, under SSP3-7.0, estimated changes in ozone concentrations are larger in the MPI-ESM-1-2-HAM (average increase of 8 μg/m^3^) than in the other four models (average increase of 4 μg/m^3^). Second, there are noticeable spatial differences in the changes in ozone concentrations in certain geographies such as in central Europe and eastern Asia across the models. These differences across models may be attributed to factors such as differences in treatment of physical and chemical interactions, grid resolution, and initial conditions for atmospheric variables and chemical species.

### Ozone-related short-term excess mortality

In the present period (2010–2014), short-term exposure to ozone concentrations above 70 μg/m^3^ accounts for 0.17% (95% empirical confidence interval [eCI]: 0.13%–0.21%) of total deaths in the 406 cities. This is equivalent to about 6,600 deaths per year (95% eCI: 2,600–10,100 deaths per year), similar to previous estimates from Vicedo-Cabrera et al.^5^ Large numbers of ozone-related deaths are found in key cities—such as 557 (95% eCI: 83–1,057) deaths per year in the Valley of Mexico, Mexico; 250 (95% eCI: 116–425) deaths per year in Los Angeles, CA, United States; 157 (95% eCI: 74–268) deaths per year in Tokyo, Japan; 138 (95% eCI: 75–202) deaths per year in Riverside, CA, United States; 121 (95% eCI: 75–212) deaths per year in Guadalajara, Mexico; 118 (95% eCI: 65–183) deaths per year in Toronto, ON, Canada; and 105 (95% eCI: 0–226) deaths per year in Taipei, Taiwan. When mortality estimates are restricted to days where ozone concentrations are above the WHO guideline of 100 μg/m^3^, health impacts are reduced to 3,600 ozone-related deaths per year. Total estimates of ozone-related acute excess mortality vary moderately (range: 5,500–7,500 deaths per year) across global chemistry-climate models in the present period, with the highest total mortality estimates produced by GFDL-ESM4 and the lowest total mortality estimates produced by CESM2.

**Figure 2:**
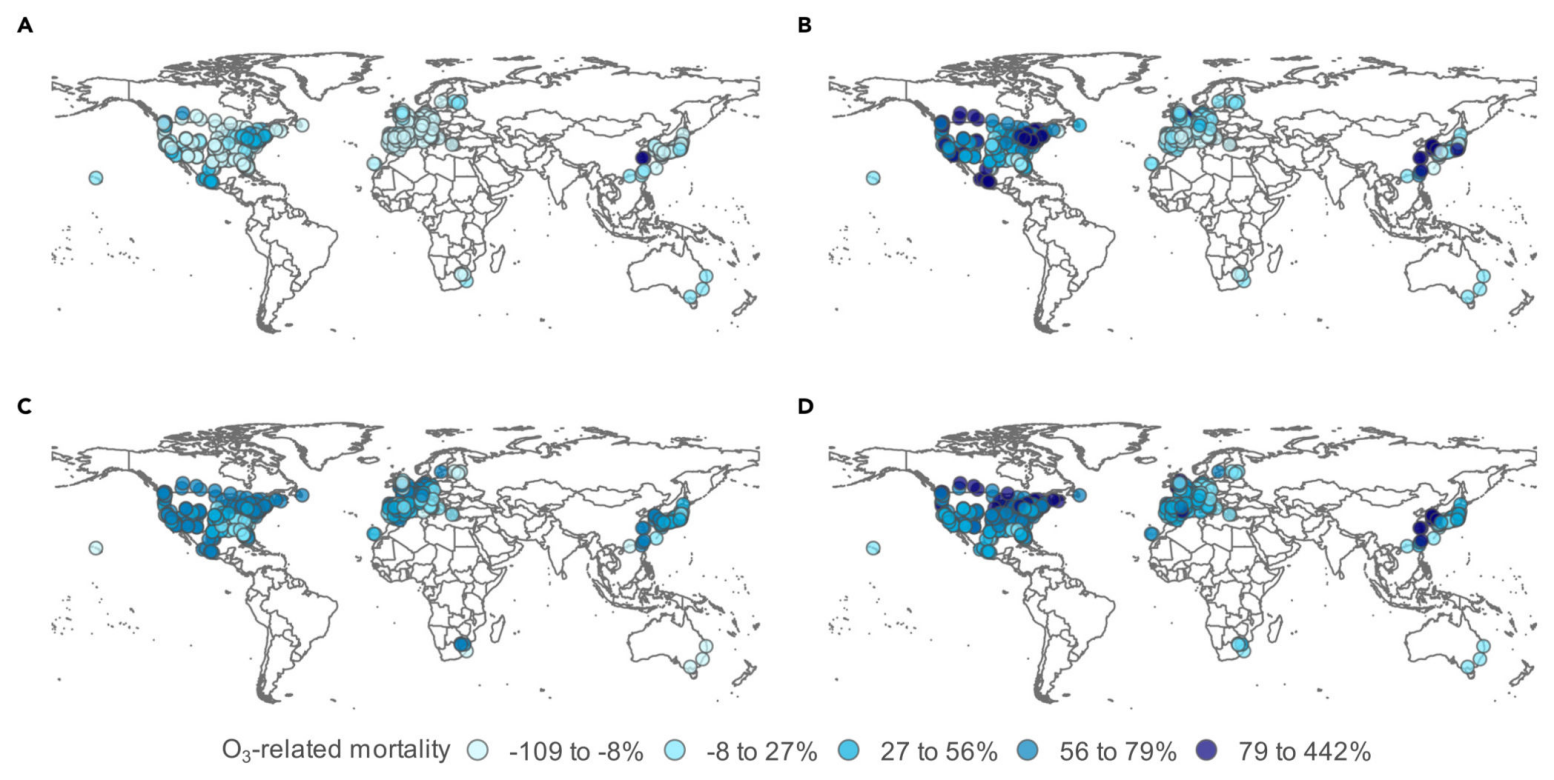
Change in O_3_-related excess mortality between present (2010–2014) and future (2050–2054) time periods in 406 locations in 20 countries or regions Absolute changes in O3-related excess mortality under (A) SSP1-2.6, (B) SSP2-4.5, (C) SSP3-7.0, and (D) SSP5-8.5.

Under SSP-1.2.6, a scenario with strong climate and air pollution controls that are aligned with the Paris Climate Agreement, ozone-related acute excess mortality is estimated to account for 0.15% (95% eCI: 0.11%–0.19%) of total deaths in the future period, with total ozone-related mortality increasing by 0.7% (95% eCI: −5% to 7%) between the present (2010–2014) and future (2050–2054) periods. Ozone-related mortality is projected to decrease in 70% of the studied cities but is notably increasing in several cities in Canada, China, Mexico, and the United States (Figure 2A; Table 1).

Under SSP-2.4.5, the middle-of-the-road scenario, ozone-related acute excess mortality is estimated to account for 0.21% (95% eCI: 0.15%–0.26%) of total deaths in the future period, with total ozone-related mortality increasing by 64% (95% eCI: 49%–82%) between the present (2010–2014) and future (2050–2054) periods. Ozone-related mortality is projected to increase in 75% of the studied locations, with large increases in cities in North America and Asia and small increases or decreases in cities in Europe and South Africa (Figure 2B; Table 1).

Under SSP-3.7.0, the regional rivalry scenario with weak climate and air pollution controls, ozone-related acute excess mortality is estimated to account for 0.21% (95% eCI: 0.16%–0.26%) of total deaths in the future period, with total ozone-related mortality increasing by 94% (95% eCI: 73%–116%) between the present (2010–2014) and future (2050–2054) periods. Increases in ozone-related excess mortality were projected in 94% of cities included in the analysis, with small to moderate increases in cities in eastern Europe, Japan, and southeastern United States and large increases in cities in all other parts of North America, northwestern Europe, China, Taiwan, and South Africa (Figure 2C; Table 1).

Under SSP-5.8.5, a scenario with weak climate controls but strong air pollution controls, ozone-related acute excess mortality is estimated to account for 0.22% (95% eCI: 0.17%–0.29%) of total deaths in the future period, with total ozone-related mortality increasing by 56% (95% eCI: 42%–87%) between the present (2010–2014) and future (2050–2054) periods. Increases in ozone-related excess mortality were projected in 94% of cities included in the analysis, with small to moderate increases in cities in China and Europe and large increases in cities in North America and all other parts of Asia (Figure 2D; Table 1).

### Drivers of changes in ozone-related excess mortality

Changes in ozone-related mortality are driven by three key factors: changes in emissions and climate, population size, and mortality rates. Here, we isolate the relative contribution of each factor in influencing changes in ozone-related mortality between present and future periods. Though national age-specific baseline mortality rates are generally projected to decline in the studied countries or regions across all SSPs, we observe an increasing proportion of older individuals between 65 and 74 years and 75 years and older (Figure S2). Across the SSPs, age-specific baseline mortality rates for individuals above 75 years are on average 24–41 times greater than individuals between 0 and 64 years old, resulting in higher overall baseline mortality rates.

**Figure 3:**
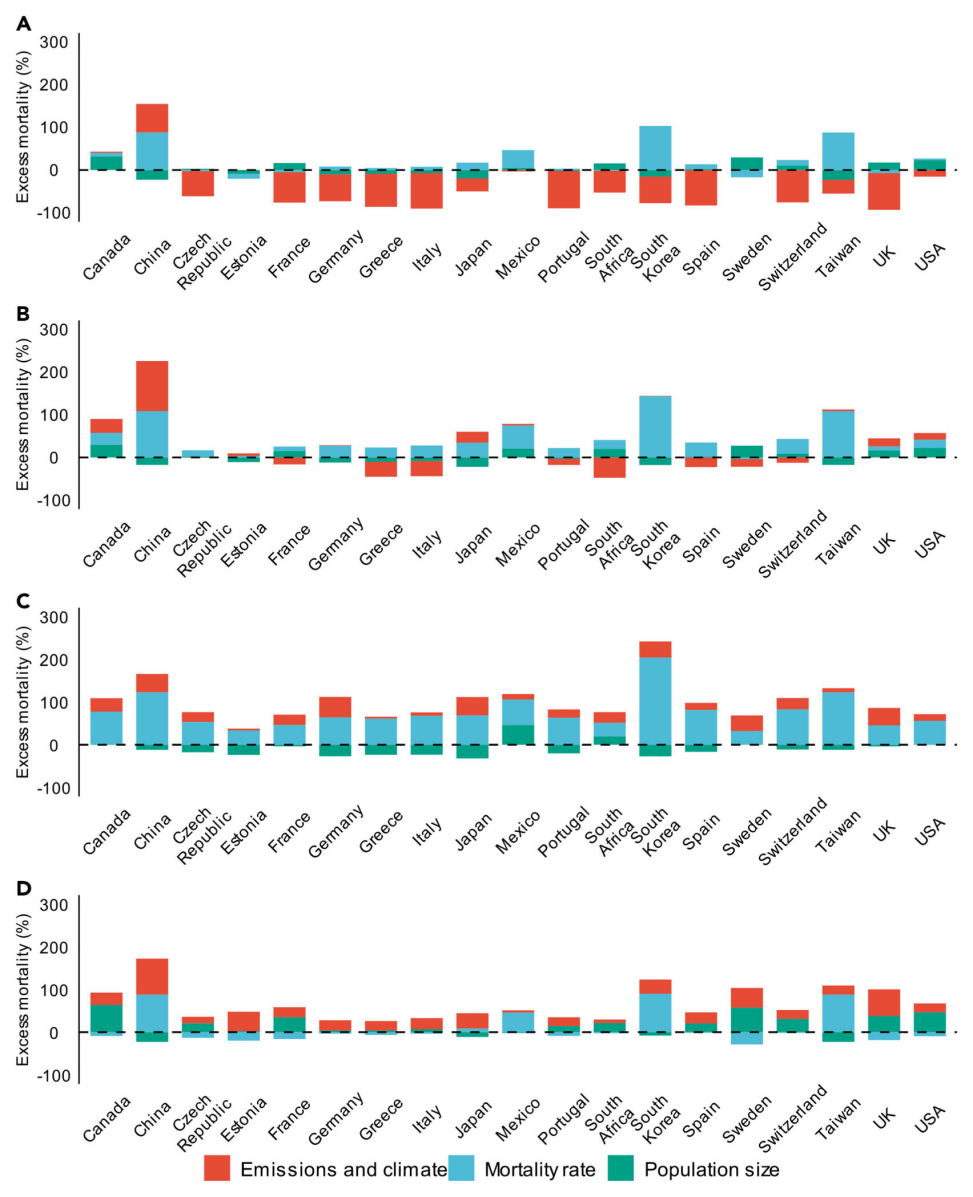
Changes in O_3_-related mortality between present (2010–2014) and future (2050– 2054) time periods allocated to changes in emissions and climate, mortality rates, and population City-level changes in O_3_-related mortality under (A) SSP1-2.6, (B) SSP2-4.5, (C) SSP3-7.0, and (D) SSP5-8.5 are aggregated to the country or region level.

Under SSP1-2.6, total ozone-related mortality is estimated to increase by 45 deaths/year (CI: −940 to 980 deaths/year) between the present period (2010–2014) and the future period (2050–2054). Changes in emissions and climate are linked to net decreases in ozone-related mortality fractions (range: −97% to −3% decrease in deaths per year) in all studied countries or regions except China (67% increase in deaths per year) and Canada (3% increase in deaths per year). Changes in population size generally increase ozone-related mortality in North America, South Africa, Sweden, France, Switzerland, Czech Republic, and Spain (range: 3%–49% increase in deaths per year) and decrease it in Asia and the remaining European countries or regions (range: −23% to −2% deaths per year). Changes in baseline mortality rates are projected to decrease ozone-related mortality in South Africa, Czech Republic, France, UK, Estonia, and Sweden (range: −17% to −1% deaths per year) and to increase it in all other countries or regions (Figure 3A).

Under SSP2-4.5, total ozone-related mortality is estimated to increase by 4,400 deaths/year (CI: 2,300–6,600 deaths/year) between the present period (2010–2014) and the future period (2050–2054). Changes in emissions and climate are linked to net increases in ozone-related mortality in Asia, North America, UK, Estonia, and Germany (range: 1%–118% deaths per year) and to net decreases in all other countries or regions (range: −48% to −0.4% deaths per year). Changes in population size generally decrease ozone-related mortality in Asia, Germany, Estonia, Greece, Italy, and Portugal (range: −22% to −3% deaths per year) and increase it in North America, South Africa, and the remaining European countries or regions (range: 1%–49% deaths per year). Changes in baseline mortality rates are projected to increase ozone-related mortality in all studied countries or regions (range: 4%–144% deaths per year) except Sweden (−4% deaths per year) (Figure 3B).

Under SSP3-7.0, total ozone-related mortality is estimated to increase by 6,200 deaths/year (CI: 3,100–9,300 deaths/year) between the present period (2010–2014) and future period (2050–2054). Changes in emissions and climate are consistently associated with net increases in ozone-related mortality (range: 4%–47% increase in deaths per year) at a country or region level. Changes in population size slightly decrease ozone-related mortality (−32% to −0.02% decrease in deaths per year) due to the decreasing population size projected in most countries or regions, except for Mexico, South Africa, and Sweden, where populations are increasing (0.5%–46% increase in deaths per year). Decreases in population size are consistently offset by increasing baseline mortality rates (32%–105%), largely driven by population aging (Figure 3C).

Under SSP5-8.5, total ozone-related mortality is estimated to increase by 3,700 deaths/year (CI: 2,000–5,800 deaths/year) between the present period (2010–2014) and the future period (2050–2054). Changes in emissions and climate are consistently linked to a net increase in ozone-related mortality (range: 5%–84% deaths per year). Changes in population size generally decrease ozone-related mortality in Asia and Mexico (range: −23% to −2% deaths per year) and increase it in all other studied countries or regions (range: 20%–57% deaths per year). Conversely, changes in baseline mortality rates generally increase ozone-related mortality in Asia and Mexico (range: 8%–90% deaths per year) and decrease it in all other studied countries or regions (range: −29% to −2% deaths per year) (Figure 3D).

## Discussion

Ground-level ozone exposure, associated with premature mortality and morbidity,^3–5^ is projected to worsen in many regions due to climate-driven meteorological shifts and changes in ozone precursor emissions.^6^ Despite this, a comprehensive estimation of future changes in ozone-related acute excess mortality across diverse geographical locations and under various climate and air quality scenarios using the most recent CMIP6 model ensembles has been lacking. Our study addresses this gap, building on the largest epidemiological study to date,^5^ by quantifying future changes in ozone-related acute excess mortality in 406 cities across 20 countries or regions. We project an increase in ozone-related deaths in all studied scenarios, ranging from 45 to 6,200 deaths/year in the studied 406 cities, accompanied by rising mortality fractions (from 0.17% to 0.21%–0.22% of total deaths), except in the scenario aligned with the Paris Climate Agreement goals (decreasing from 0.17% to 0.15% of total deaths).

Our analysis projects about 6,600 ozone-related deaths per year in the 406 cities in the present period, consistent with a previous study that estimated ozone-related deaths for the same MCC network.^5^ This work advances the prior MCC study by estimating future changes in ozone-related acute excess mortality, employing multi-model ensembles under CMIP6 under four distinct SSP scenarios to provide a novel and comprehensive projection of future ozone-related mortality burden. Under SSP1-2.6, SSP2-4.5, SSP3-7.0, and SSP5-8.5, ozone-related acute excess mortality in the studied 406 cities is estimated to increase by 45, 4,400, 6,200, and 3,700 deaths/year, respectively. These future estimates are broadly consistent with past research that show small to large increases in health impacts under scenarios with high global warming and emissions of ozone precursors and moderate decreases to small increases in health impacts under scenarios with low global warming and emissions of ozone precursors.^8–10^ However, these studies cannot be directly compared, as they were conducted over different geographical boundaries, climate scenarios, socioeconomic pathways, and threshold concentrations. We address gaps of previous studies that investigated future trends in ozone-related acute excess mortality by expanding our analysis to a broader geographical scope and considering a wider range of climate and air quality scenarios.

Previous studies that examined future ozone-related acute excess mortality were often constrained to long-term exposure metrics linked to specific causes (e.g., respiratory mortality), narrow geographical scopes, limited climate and air quality scenarios, and location-specific ozone-mortality relationships that have been generalized to wider populations. Our study builds on this prior work in several fundamental ways. First, we concentrate on short-term ozone-mortality relationships that encompass a broader spectrum of mortality causes beyond respiratory and cardiovascular disease. Second, we significantly broaden the geographic scope, analyzing 406 cities in 20 countries or regions. Third, we estimate health impacts from ground-level ozone concentrations under four SSP scenarios, which consider a wide array of climate and air pollution controls. Fourth, we incorporate country- or region-specific ozone-mortality relationships derived from a recent MCC study^5^ to address the uncertainty linked to location-specific parameters in air pollution health impact assessments, as underscored previously.^11^ These enhancements produce a more accurate estimate of the overall health impacts of short-term exposure to ozone, which can inform the design of more nuanced health policies.

Four primary limitations are encountered in our study: first, while our analysis uses baseline mortality rates that are adjusted for changes in demographic composition, the ozone-mortality relationships we utilize are not age specific. Performing age-stratified analysis may be key to more fully accounting for ozone-related health burdens given the increased vulnerability

of older populations to air pollution exposure.^12^ Indeed, our study found that changes in baseline mortality rates, largely influenced by changes in age demographics, are often the biggest driver of changes in ozone-related deaths and underscore the need to conduct further analysis that uses age-specific ozone-mortality relationships. Second, our approach of using the 70 μg/m^3^ threshold based on a previous health impact assessment^5^ also does not incorporate the ozone concentration changes below this threshold. Further epidemiological studies are needed to examine if there are sufficient evidence on the adverse ozone effects on health below this threshold. Third, though the CMIP6 project includes future ozone projections of at least 26 models across the four SSPs, most archived ozone fields at a coarser temporal resolution (e.g., monthly) that is not suitable to evaluate short-term exposure to ozone. Consequently, the analysis is constrained to simulations with 1–5 available global chemistry-climate models, depending on the SSP being modeled. Fourth, although our analysis largely expands on the geographical scope of previous studies, the study cannot be considered a true global analysis, as it is limited to the 406 cities within the MCC network for which there are observed ozone concentrations and ozone-mortality relationships.

Our findings underscore the need for more stringent air quality regulations, given that current ozone standards in many of the studied countries or regions exceed 70 μg/m^3^, a threshold at which concentrations are largely attributed to non-anthropogenic sources and ozone-mortality relationships are established.^5^ For instance, in the United States, the Environmental Protection Agency has set the primary and secondary 8-h standard to approximately 137 μg/m^3^.^13^ Similarly, the European Union’s Air Quality Directive prescribes maximum daily 8-h average concentrations of 120 μg/m^3^.^14^ Canada’s ambient air quality standards are established at 122 μg/m^3^,^15^ while in Korea, the Ministry of Environment regulates ozone concentrations are set to 118 μg/m^3^.^16^ Higher thresholds of daily maximum 8-h average concentrations are implemented in China at 160 μg/m^317^ and in Mexico at 137 μg/m^3^.^18^ Our study highlights the need for more rigorous ozone standards. Beyond mitigating ozone-related acute excess mortality, the implementation of stricter air quality regulations will likely yield additional benefits in terms of reducing long-term ozone-related mortality and conferring climate benefits.

## Experimental Procedures

### Resource availability

#### Lead contact

Further information and requests for resources should be directed to and will be fulfilled by the lead contact, Dr. Kai Chen.

#### Materials availability

This study did not generate new unique materials.

### Study design

This analysis estimated future excess mortality from short-term exposure to ozone in 406 cities in 20 countries or regions. The 406 cities in 20 countries or regions are distributed over North America, Europe, Asia, Australia, and Africa.

### Historical ozone observations and baseline mortality

Historical ambient ozone observations and baseline mortality counts for 406 cities in 20 countries or regions were obtained from the MCC Collaborative Research Network. Daily observations of historical ozone concentrations from at least one monitor were obtained for each city and presented in terms of maximum daily 8-h average concentrations. For cities that had more than one monitor, historical ozone concentrations were computed as the average of the observed concentrations from all available monitors. It is also important to note that ozone data in Japan were derived from the measurements of photochemical oxidant, which is primarily ozone (≥90%), followed by other species such as peroxy acetyl nitrate (PAN), hydrogen peroxide, and organic hydroperoxides. Baseline mortality counts were measured in terms of daily all-cause deaths for cities located in Canada, the Czech Republic, Estonia, France, Germany, Greece, Italy, Japan, Mexico, Portugal, South Africa, South Korea, Sweden, Taiwan, UK, and the United States; daily deaths due to non-external causes for cities in Australia, China, and Spain; and daily deaths due to non-external causes other than unintentional injuries for cities in Switzerland.

Historical ozone observations and baseline mortality counts were available in largely overlapping periods between January 1, 1985, to December 31, 2015, across the 406 cities. To minimize computational demands of the analysis, we utilized data from the last three full and consecutive years for each city to bias correct ozone concentrations and estimated ozone-related acute excess mortality in present (2010–2014) and future (2050–2054) periods. The mid-century time frame was selected to represent the future period to ensure the relevancy of results to present-day policy formation.

### Present and future ozone projections

We obtained global ozone simulations performed with five global chemistry-climate models from the Aerosols and Chemistry Model Intercomparison Project (AerChemMIP) under the CMIP6. These five chemistry-climate models include the Community Earth System Model v.2 (CESM2), the EC-Earth3-AerChem, Geophysical Fluid Dynamics Laboratory Earth System Model v.4 (GFDL-ESM4), the Max Planck Institute Earth System Model (MPI-ESM1-2-HAM), and the UK Earth System Model (UKESM-1-0-LL). Surface ozone concentrations were available at an hourly temporal scale, which we used to calculate MDA8 ozone concentrations. We then applied a constant scaling factor of (1 ppb = 1.96 μg/m^3^) to convert the molar mixing ratios to mass densities. Further information on each of the models can be found in Table S3. For this analysis, historical simulations for the years 2010–2014 were utilized to represent the present period, and simulations corresponding SSP1-2.6, SSP2-4.5, SSP3-7.0, and SSP5-8.5 for the years 2050–2054 were utilized to represent the future period. While it would be ideal to model time periods exceeding 5 years, our analysis is limited to a 5-year time frame due to the constraints in computational resources. Different numbers of models are available for each SSP, and even when a model simulated multiple SSPs, they provided different numbers of ensemble members. Despite this limitation, we chose to use ozone projections from all available models and ensemble members in our study to maximize the number of models we could include.

The SSPs were developed for the CMIP6 in 2017^19^ and thus do not explicitly incorporate climate and air quality policies or market changes (e.g., electric vehicles) that have occurred since then. Nevertheless, these changes fall within the wide range of possible future spanned by the SSPs, which were designed to cover different possible future socioeconomic pathways.^19^

As applied in a previous study,^8^ we performed a bias-correction technique on the modeled ozone concentrations from the five global chemistry-climate models to obtain ozone concentrations for each of the 406 cities in both present and future periods. Bias correction techniques increase the utility of publicly available models, which are still imperfect mathematical representations of the Earth’s climate system. Biases in climate models arise due to simplified representations of atmospheric chemistry and physics, coarse spatial resolution, and incomplete understanding of the global climate system.^20^ A recent global study demonstrated that after bias correction using a similar quantile mapping approach, the data accuracy of ozone concentrations improved pronouncedly, with increases in the correlation coefficient and decreases in the root-mean-square error between the observational and modeled data.^21^

To perform the bias-correction step, we first computed monthly biases between the observed ozone concentrations and modeled ozone concentrations. Specifically, monthly biases were computed for each quantile of the modeled ozone concentrations within the boundaries of the grid cell from each chemistry-climate model. Assuming historical monthly biases persist throughout time, we then corrected for these biases in present and future time periods.

### Changes in baseline mortality counts

We computed daily baseline mortality counts in each of the 406 cities in present and future periods as follows:

(Equation 1) $${\text{M}}_{\text{p}/\text{f}}={\text{M}}_{\text{h}}\times {\text{ΔY}}_{\text{b}}\times \text{ΔPOP},$$

where M_p/f_ is the city-level daily baseline mortality count in the present or future period, M_h_ is the city-level daily baseline mortality count corresponding to the last full and consecutive 3 years of available historical data, ΔY_b_ is the change in country-or region-level annual baseline mortality rate between the historical period and the present or future period, and ΔPOP is the change in country- or region-level population between the historical period and the present or future period. We trimmed the daily baseline mortality count to the last full and consecutive 3 years from the MCC network to be consistent with the criteria we used for the observed ozone concentration data in the bias correction stage.

Country- or region-level projections of baseline mortality rates and population across the four SSPs were obtained from the SSP project, and historical estimates of baseline mortality and population were obtained from the United Nations’ World Population Prospects 2019 Report.

### Health impact assessment

We estimated the daily ozone-related excess mortality in present and future periods using the attributable fraction (AF) approach. The AF is defined as the share of the baseline mortality that is attributable to short-term exposure and is computed as follows:

(Equation 2) $$\text{AF}=1-exp^{-\beta \times C},$$

where *C* is the difference between the city-level maximum daily 8-h average concentration for ozone and the maximum background levels of 70 μg/m^3^ and *β* corresponds to the logarithm of the relative risk at ozone concentration *C*. In this analysis, country-or region-specific exposure-response functions are obtained from Vicedo-Cabrera et al.^5^ for ozone concentrations above maximum background levels. Exposure-response functions in this study were obtained through a two-stage time-series analysis of about 45 million deaths. In the first stage, the study runs separate time-series regression models to obtain city-specific ozone-mortality risks. In the second stage, the study pools the city-specific estimates through a meta-analysis to derive the best linear unbiased predictions of the risks at a country or region level. We prioritize using country- or region-specific ozone-mortality relationships in our study, as Vicedo-Cabrera et al. found that these relationships were spatially heterogeneous (range: 1.0008–1.0035 relative risk per 10 μg/m^3^ increase in ozone).^5^ These spatial differences in ozone-mortality relationships may be driven by local conditions, including concentrations and sources of ozone precursors, and population characteristics—which we would not be able to capture by extrapolating a single ozone-mortality relationship to the entire globe.

For global chemistry-climate models that have multiple ensemble members, and thus multiple estimates of concentrations for the same period and city, we use the average of all bias-corrected concentrations across all available ensemble members.

The attributable daily deaths due to short-term ozone exposure in each city and period are then calculated as follows:

(Equation 3) $$ADD=M_{p/f}\times \text{AF},$$

where *ADD* is the estimated city-level number of daily deaths attributed in present and future periods and *M*_*p/f*_ and AF are as defined above. Annual attributable mortality is then computed by summing the *ADD* throughout the year in each period. Lastly, we computed the absolute and percentage changes in ozone-related excess mortality between present and future periods.

Uncertainty in mortality estimates was quantified using Monte Carlo simulations that incorporated uncertainty from coefficients in the exposure-response functions and inter-model variability. For absolute mortality estimates, we derived CIs at a 90% confidence level from the distribution across 1,000 coefficient samples, assuming a normal distribution, for each of the five global chemistry-climate models. To derive CIs for changes in mortality over time, we subtract the mean mortality estimate in the present period from the lower-or upper-bound mortality estimate in the future period.

As a sensitivity test, we compared the ozone-related excess mortality in the present period using both the raw simulated ozone concentrations and bias-corrected ozone concentrations. To minimize computational requirements, we limit the sensitivity test to the four cities that are associated with the highest ozone-related mortality in the main analysis. Across the four cities, ozone-related excess mortality differed by −11% to 550% between the two ozone datasets (Table S2); therefore, we elected to limit our main analysis to the bias-corrected ozone concentrations.

Changes in estimated ozone-related mortality between present and future periods can be attributed to three factors—emissions and climate, population size, and mortality rates. We decomposed the impacts of each of these three factors by first running a scenario with changing emissions and climate and fixed population and mortality rates to isolate the impact of emissions and climate. All else held constant, changes in ozone-related mortality are directly proportional to changes in population size and changes in mortality rates and could be readily isolated without further computation.

## Supplementary Material

Supplemental material

## Figures and Tables

**Table 1:** Change in O_3_-related mortality between present (2010–2014) and future (2050–2054) time periods under each SSP

Country or region	Number of cities	Mean change in O**_3_**-related mortality, deaths/year (CI)					Change in O**_3_**-related mortality, % (% CI)			
		SSP1-2.6	SSP2-4.5	SSP3-7.0	SSP5-8.5		SSP1-2.6	SSP2-4.5	SSP3-7.0	SSP5-8.5
Australia	3	0 (0−0)	0 (0−0)	0(0−0)	0 (0−0)		–	–	–	–
Canada	26	157 (101−215)	415(271−568)	441 (284−603)	323 (209−454)		46 (30−64)	120(78−164)	132 (85−181)	93(60−131)
China	3	59 (19−98)	115(37−188)	104 (32−178)	80(26−136)		142 (44−233)	274 (88−448)	179(56−310)	168 (54−285)
Czech Republic	1	−19 (−33 to −5)	5 (2−9)	17 (5−28)	6(2−11)		−59 (−100 to −16)	16(5−28)	54 (15−93)	21 (6−37)
Estonia	4	0 (0−0)	0 (0−0)	0(0−0)	0 (0−0)		−98 (−157 to −35)	−2 (−5 to 6)	4(1−31)	19(6−37)
France	18	−78 (−122 to −32)	8(3−19)	98 (40−153)	61 (25−104)		−68 (−107 to −28)	6(2−16)	75(31−118)	40 (17−68)
Germany	12	−63 (−97 to −29)	13(6−21)	78 (37−122)	28 (12−46)		−64 (−100 to −30)	14(6−22)	77(36−121)	24 (11−40)
Greece	1	−31 (−69 to 0)	−12 (−26 to 0)	12 (1−27)	9(0−21)		−79 (−176 to 0)	−28 (−62 to 0)	17 (2−63)	18(0−44)
Italy	13	−118(−203 to −35)	−35 (−61 to −9)	59 (17−102)	45 (13−85)		−83(−144 to −24)	−25 (−43 to −7)	37(11−67)	30 (9−57)
Japan	43	−339(−489 to −201)	318(187−470)	614 (366−897)	320 (185−499)		−35 (−50 to −20)	32 (19−47)	64 (38−93)	31 (18−48)
Mexico	8	378(90−661)	800 (183−1,409)	1,385 (329−2,433)	415(87−770)		44 (10−77)	93 (21−164)	160(39−288)	51 (11−94)
Portugal	6	−26 (−54 to 0)	0 (0−3)	18 (1−37)	8 (0−17)		−88 (−185 to 0)	1 (0−9)	45(4−116)	26 (0−56)
South Africa	4	−43 (−62 to −24)	−23 (−34 to −13)	105(57−158)	30 (15−59)		−45 (−64 to −24)	−24(−35 to −13)	97(53−146)	28 (14−55)
South Korea	7	−30 (−49 to −10)	86(29−142)	188(65−310)	116(38−193)		−35 (−58 to −12)	102 (34−167)	204 (71−339)	133(43−221)
Spain	47	−32 (−83 to 0)	2 (0−5)	29 (1−74)	19(0−50)		−81 (−212 to 0)	5(0−13)	41 (3−193)	49(0−129)
Sweden	1	−4 (−7 to −1)	0 (0−0)	4(1−6)	4(1−6)		−91 (−151 to −29)	1 (0−7)	80(25−134)	64(20−109)
Switzerland	8	−20 (−33 to −6)	8(2−13)	30 (9−52)	16(5−27)		−70 (−119 to −22)	28 (9−47)	104(33−181)	56 (17−95)
Taiwan	3	−4 (−8 to 0)	180 (7−335)	283 (19−529)	147(6−279)		−2 (−3 to 0)	79 (3−146)	106(8−216)	76(3−145)
UK	15	−17(−21 to −12)	10(8−14)	30 (22−38)	18(13−26)		−85(−108 to −64)	51 (38−68)	97(72−123)	82 (60−117)
United States	183	273 (183−364)	2,516 (1,687−3,408)	2,689 (1,833−3,579)	2,076 (1,362−2,973)		8(5−10)	69 (46−93)	80(55−106)	60 (40−87)

City-level changes in O_3_-related mortality are aggregated to the country or region level. Values are rounded to the nearest whole number.

## Data Availability

The projected data on temperature and ozone concentration can be obtained from the CMIP6 database (https://esgf-node.llnl.gov/search/cmip6/). The projected population data can be obtained from the Socioeconomic Data and Applications Center, Global 1-km Downscaled Population Base Year and Projection Grids Based on the SSPs, v1.01 (2000 – 2100): https://doi.org/10.7927/q7z9-9r69. The historical baseline mortality and population data can be obtained from the United Nations’ World Population Prospects 2019 Report (https://population.un.org/wpp/Download/Standard/MostUsed/). Code used to generate the results are publicly available on Github (https://github.com/CHENlab-Yale/MCC_FutureO3). Any additional information required for reanalyzing the data reported in this paper is available from the lead contact upon reasonable request.

## In brief

In brief Climate change and changes in emissions of ozone precursors will elevate future ground-level ozone in many parts of the world—the short-term exposure to which is linked to increased mortality. We explored future ozone-related acute excess mortality trends in 406 cities across 4 climate scenarios. Ozone-related mortality fractions increase in all scenarios except the one aligned with the Paris Climate Agreement. This underscores the urgent need for stricter air quality regulations globally.

## Science for Society

Ground-level ozone—a product of reactions between other pollutants in the atmosphere—can trigger coughing and shortness of breath, worsen asthma, and cause damage to airways. Short-term exposure to ground-level ozone can lead to respiratory and cardiovascular disease and has been linked to higher rates of premature death. Climate change and changes in the atmospheric concentrations of ozone precursor pollutant are projected to increase ground-level ozone in many parts of the world. However, in the absence of a firm understanding of how ozone-related mortality will change in different regions, and under different climate, and air quality scenarios, our ability to create effective public health policies is limited. Our research indicates that the total deaths related to short-term ozone exposure will increase in 406 cities around the world under all studied scenarios, but the scenario aligned with the goals of the Paris Climate Agreement. These findings highlight the urgent need for stricter air quality regulations, as the current standards in many countries are not sufficient to address this growing threat.

## Highlights

- Total ozone-related deaths increase across all studied scenarios
- Ozone-related mortality fractions rise in all but the Paris Agreement-aligned scenario
- Inadequate standards across countries necessitate tougher air quality controls
