# Supplementary material for: Ozone-related acute excess mortality projected to increase in the absence of climate and air quality controls consistent with the Paris Agreement
Source: One Earth. Author manuscript; Available in PMC 2024 Feb 28. (PMC7615682; doi:10.1016/j.oneear.2024.01.001)
Supplement: Supplemental material [file EMS194248-supplement-Supplemental_material.pdf]

One Earth, Volume 7

Supplemental information

Ozone-related acute excess mortality projected to increase in the absence of climate and air quality controls consistent with the Paris Agreement

Nina G.G. Domingo, Arlene M. Fiore, Jean-Francois Lamarque, Patrick L. Kinney, Leiwen Jiang, Antonio Gasparrini, Susanne Breitner, Eric Lavigne, Joana Madureira, Pierre Masselot, Susana das Neves Pereira da Silva, Chris Fook Sheng Ng, Jan Kyselý, Yuming Guo, Shilu Tong, Haidong Kan, Aleš Urban, Hans Orru, Marek Maasikmets, Mathilde Pascal, Klea Katsouyanni, Evangelia Samoli, Matteo Scortichini, Massimo Stafoggia, Masahiro Hashizume, Barrak Alahmad, Magali Hurtado Diaz, César De la Cruz Valencia, Noah Scovronick, Rebecca M. Garland, Ho Kim, Whanhee Lee, Aurelio Tobias, Carmen Íñiguez, Bertil Forsberg, Christofer Åström, Martina S. Ragettli, Yue Leon Guo, Shih-Chun Pan, Valentina Colistro, Michelle Bell, Antonella Zanobetti, Joel Schwartz, Alexandra Schneider, Ana M. Vicedo-Cabrera, and Kai Chen

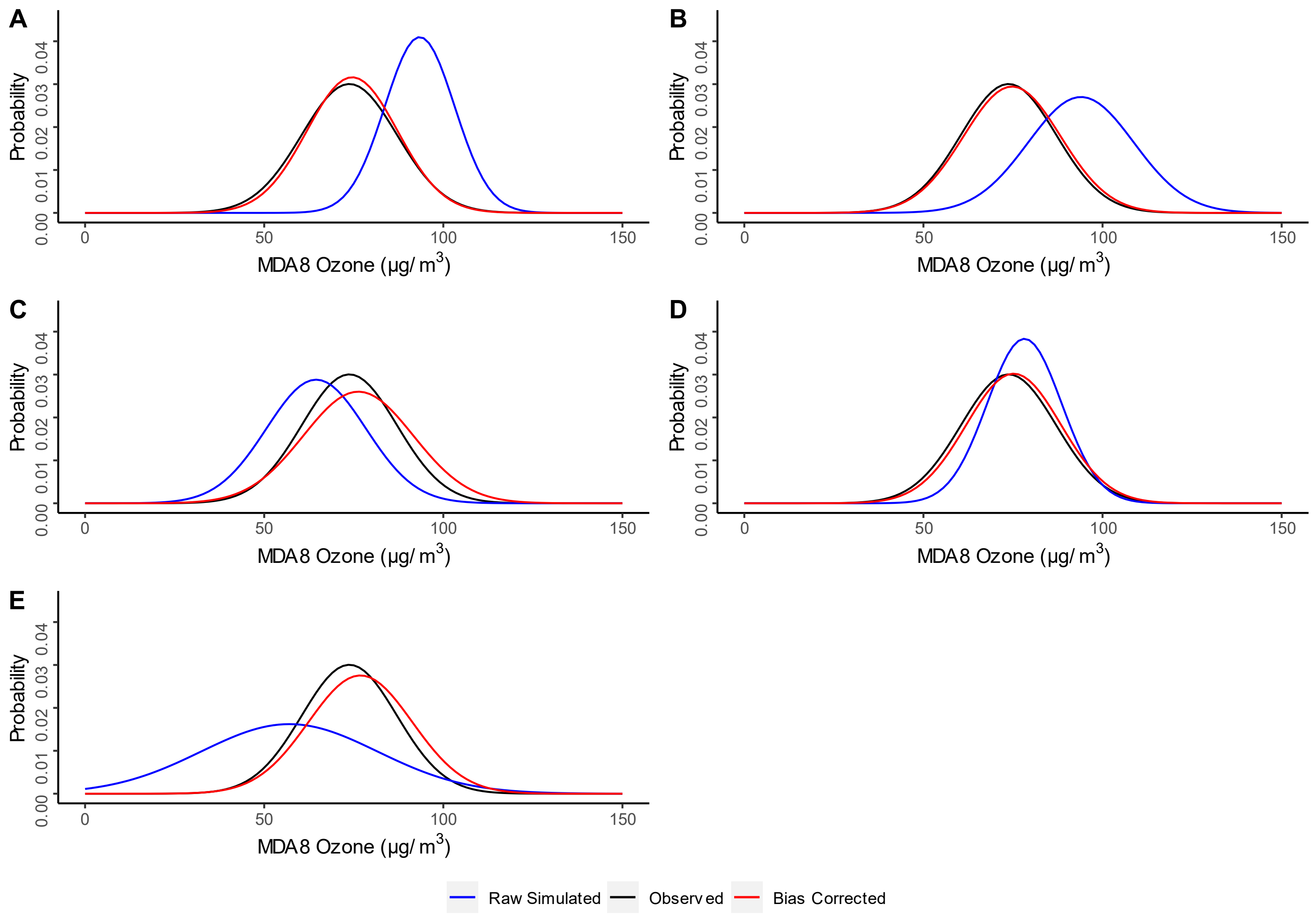

Fig S1. Raw simulated, observed, and bias corrected O_3 concentrations averaged over the 406 cities for each global chemistry-climate model in the present period. (A) CESM2, (B) EC-Earth3-AerChem, (C) GFDL-ESM4, (D) MPI-ESM-1-2-HAM, and (E) UKESM-1-0-LL.

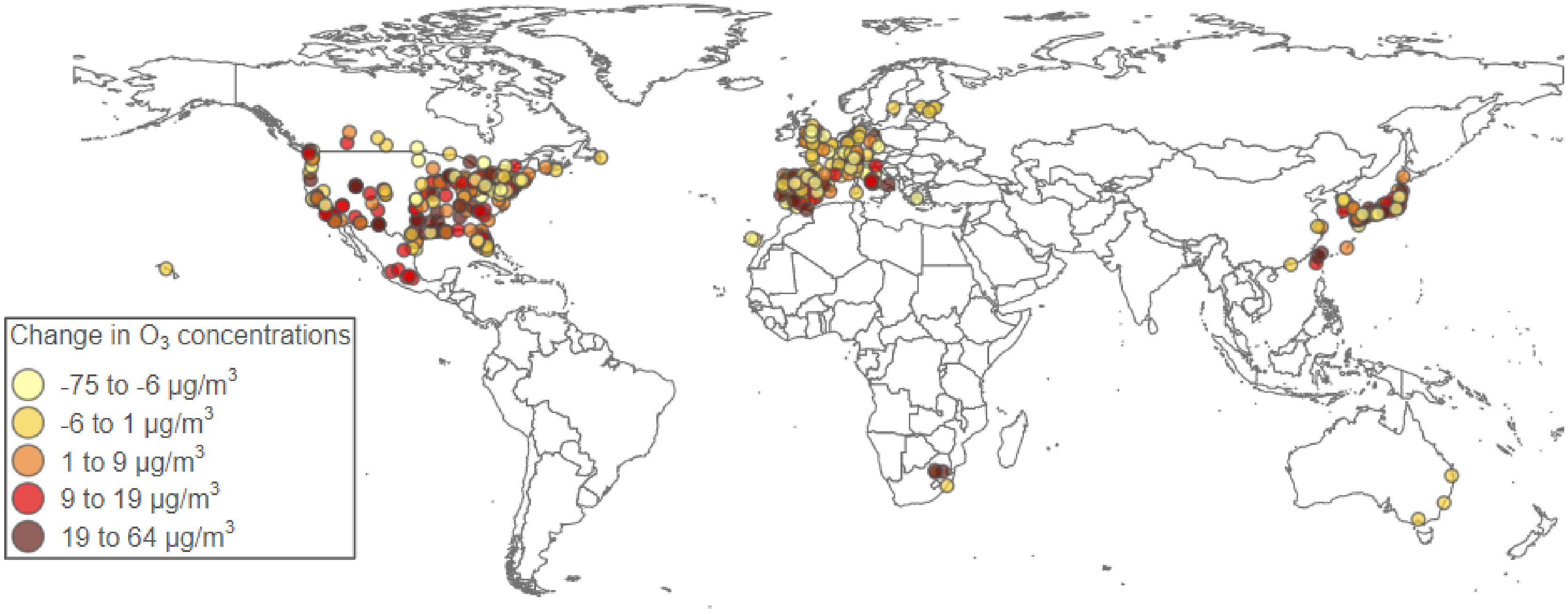

Fig S2. O_3 bias over the 406 cities average across the global-chemistry climate models in the present period. Global chemistry-climate models include CESM2, EC-Earth3-AerChem, GFDL-ESM4, MPI-ESM-1-2-HAM, and UKESM-1-0-LL.

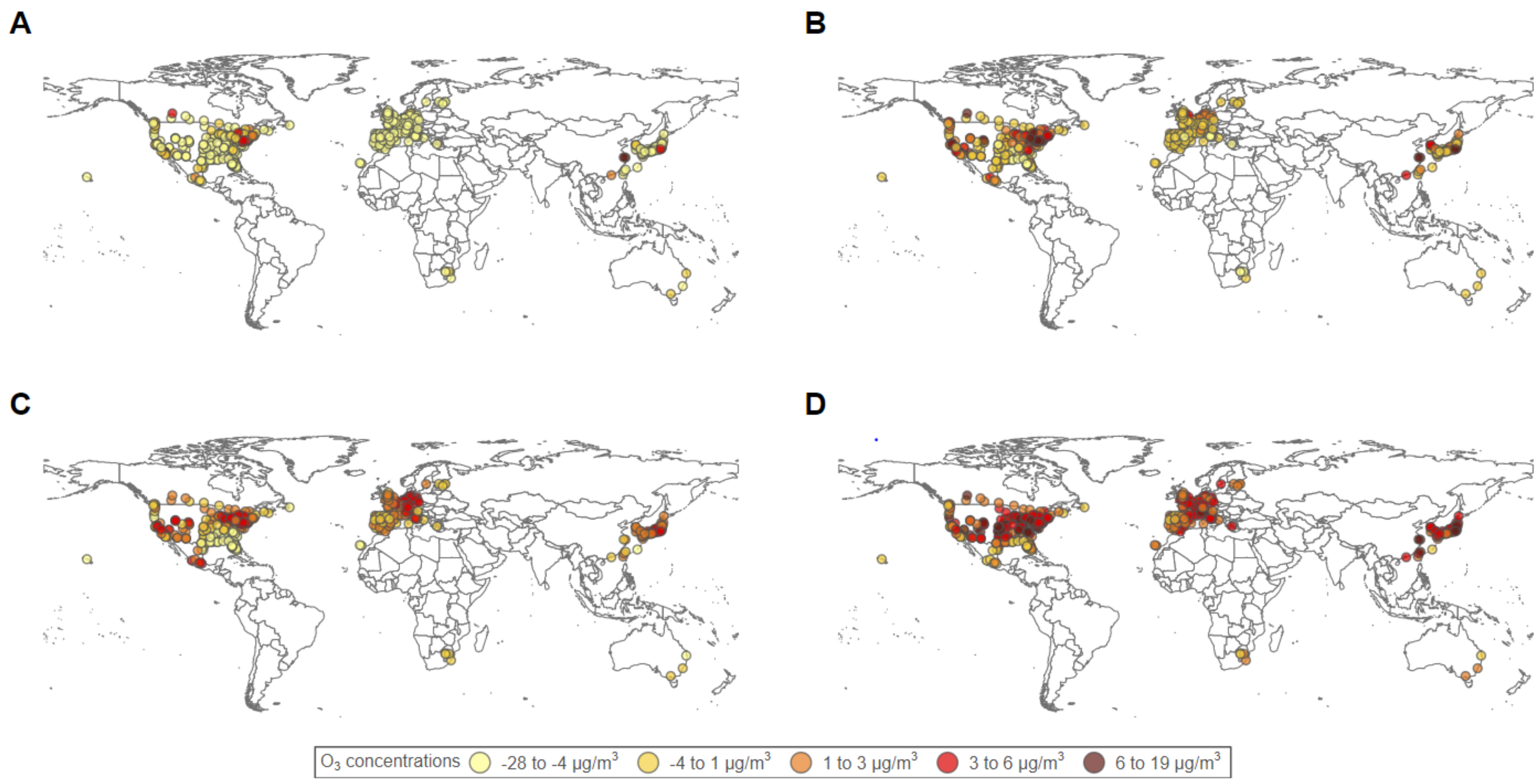

Fig S3. Absolute change in MDA8 O_3 concentration at 406 locations in 20 countries between the present (2010-2014) and future (2050-2054) time periods. (A) absolute change in O_3 concentrations under SSP 1-2.6, (B) absolute change in O_3 concentrations under SSP 2-4.5, (C) absolute change in O_3 concentrations under SSP 3-7.0, and (D) absolute change in O_3 concentrations under SSP 5-8.5. O_3 concentration is the maximum daily 8-hour average.

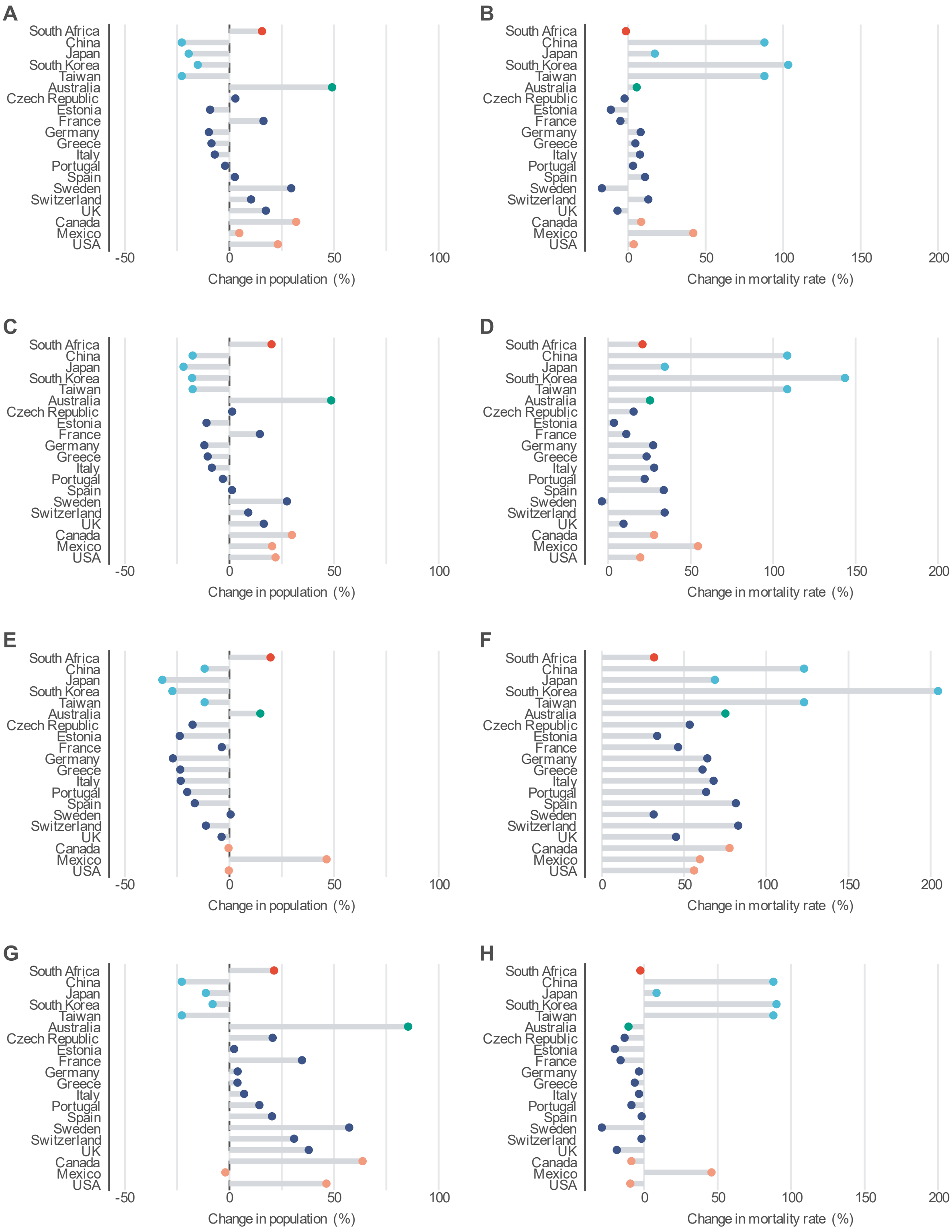

Fig S4. Change in population and mortality rates in 20 countries. Change in national population under (A) SSP 1-2.6, (C) SSP 2-4.5, (E) SSP 3-7.0, and (G) SSP 5-8.5. Change in national mortality rates under (B) SSP 1-2.6, (D) SSP 2-4.5, (F) SSP3-7.0, and (H) SSP 5-8.5.

Table S1. Change in O_3 concentrations between present (2010-2014) and future (2050-2054) time periods. City-level changes in O_3 concentrations are aggregated to the country level and rounded to the nearest whole number.

		O_3 concentrations ($\mu g/m^3$)				
**Country**	**Number of cities**	**Present data**	**SSP 1-2.6**	**SSP 2-4.5**	**SSP 3-7.0**	**SSP 5-8.5**
**Australia**	3	34	29	33	36	35
**Canada**	26	81	84	88	87	89
**China**	3	58	68	69	63	63
**Czech Republic**	1	75	58	76	82	79
**Estonia**	4	57	50	56	60	59
**France**	18	70	53	66	75	75
**Germany**	12	62	52	63	71	68
**Greece**	1	74	54	69	78	81
**Italy**	13	72	49	66	79	79
**Japan**	43	80	77	87	88	90
**Mexico**	8	133	132	135	141	131
**Portugal**	6	76	59	73	79	78
**South Africa**	4	78	73	73	81	81
**South Korea**	7	69	63	72	74	75
**Spain**	47	73	56	71	77	77
**Sweden**	1	61	53	62	65	67
**Switzerland**	8	74	54	70	82	78
**Taiwan**	3	109	95	108	113	107
**UK**	15	59	47	59	64	61

**USA**	183	84	85	92	89	92

Table S2. O_3-related mortality by global climate model in 4 cities in the present (2010-2014) period.

		O_3-related mortality (deaths/yr)	
**Global climate model**	City	**Raw simulated O_3**	**Bias corrected O_3**
CESM2	Valley of Mexico, Mexico	323	535
EC-Earth3-AerChem	Valley of Mexico, Mexico	67	530
GFDL-ESM4	Valley of Mexico, Mexico	11	614
MPI-ESM-1-2-HAM	Valley of Mexico, Mexico	28	584
UKESM-1-0-LL	Valley of Mexico, Mexico	0	524
CESM2	Los Angeles, USA	253	223
EC-Earth3-AerChem	Los Angeles, USA	227	320
GFDL-ESM4	Los Angeles, USA	31	303
MPI-ESM-1-2-HAM	Los Angeles, USA	227	178
UKESM-1-0-LL	Los Angeles, USA	0	225
CESM2	Tokyo, Japan	203	133
EC-Earth3-AerChem	Tokyo, Japan	312	151
GFDL-ESM4	Tokyo, Japan	218	144
MPI-ESM-1-2-HAM	Tokyo, Japan	82	162
UKESM-1-0-LL	Tokyo, Japan	63	193
CESM2	Riverside, USA	118	132
EC-Earth3-AerChem	Riverside, USA	97	123
GFDL-ESM4	Riverside, USA	6	157
MPI-ESM-1-2-HAM	Riverside, USA	105	124
UKESM-1-0-LL	Riverside, USA	0	151

Table S3. Global climate model and ensemble members.

Global climate model	Scenario	Ensemble member	Citation
CESM2	Historical	ic1 – 001, ic1 – 002, ic1 – 003, ic1 – 004, ic2 – 001, ic2 – 002, ic2 – 003, ic2 – 004, ic3 – 001, ic3 – 002, ic3 – 003, ic3 – 004, ic4 – 001, ic4 – 002, ic4 – 003, ic4 – 004	[1]
CESM2	SSP 3-7.0	001, 002, 003, 004	[2]
EC-Earth3-AerChem	Historical	r1i1p1f1, r4i1p1f1	[3]
EC-Earth3-AerChem	SSP 3-7.0	r1i1p1f1, r4i1p1f1	[4]
GFDL-ESM4	Historical	r1i1p1f1	[5]
GFDL-ESM4	SSP 1-2.6	r1i1p1f1	[6]
GFDL-ESM4	SSP 2-4.5	r2i1p1f1, r3i1p1f1	[7]
GFDL-ESM4	SSP 3-7.0	r1i1p1f1	[8]
MPI-ESM-1-2-HAM	Historical	r1i1p1f1, r2i1p1f1, r3i1p1f1	[9]
MPI-ESM-1-2-HAM	SSP 3-7.0	r1i1p1f1, r2i1p1f1, r3i1p1f1	[10]
UKESM-1-0-LL	Historical	r1i1p1f2	[11]
UKESM-1-0-LL	SSP 1-2.6	r1i1p1f2	[12]
UKESM-1-0-LL	SSP 2-4.5	r1i1p1f2	[13]
UKESM-1-0-LL	SSP 3-7.0	r1i1p1f2	[14]
UKESM-1-0-LL	SSP 5-8.5	r1i1p1f2	[15]

References

1. Fiore, A. M., Hancock, S., Lamarque, J. F., Correa, G., Chang, K. L., Ru, M., Cooper, O., Gaudel, A., Polvani, L., Sauvage, B., and Ziemke, J. (2022). Understanding recent tropospheric ozone trends in the context of large internal variability: A new perspective from chemistry-climate model ensembles. Environ. Res.: Climate *1*, 025008.
2. Personal communication with Dr. Jean-Francois Lamarque.
3. EC-Earth-Consortium EC-Earth3-AerChem model output prepared for CMIP6 CMIP historical (2020). Version 20200624 (Earth System Grid Federation). https://doi:10.22033/ESGF/CMIP6.4701.
4. EC-Earth-Consortium EC-Earth3-AerChem model output prepared for CMIP6 ScenarioMIP ssp370 (2020). Version 20220630 (Earth System Grid Federation). https://doi.org/10.22033/ESGF/CMIP6.4885
5. Krasting, J.P., John, J.G., Blanton, C., McHugh, C., Nikonov, S., Radhakrishnan, A., Rand, K., Zadeh, N.T., Balaji, V., Durachta, J., et al. (2018). NOAA-GFDL GFDL-ESM4 model output prepared for CMIP6 CMIP historical. Version 20190726 (Earth System Grid Federation). https://doi.org/10.22033/ESGF/CMIP6.8597.
6. John, J.G., Blanton, C., McHugh, C., Radhakrishnan, A., Rand, K., Vahlenkamp, H., Wilson, C., Zadeh, N.T., Dunne, J.P., et al. (2018). NOAA-GFDL GFDL-ESM4 model output prepared for CMIP6 ScenarioMIP ssp126. Version 20221210 (Earth System Grid Federation). https://doi.org/10.22033/ESGF/CMIP6.8684.
7. John, J.G., Blanton, C., McHugh, C., Radhakrishnan, A., Rand, K., Vahlenkamp, H., Wilson, C., Zadeh, N.T., Dunne, J.P., et al. (2018). NOAA-GFDL GFDL-ESM4 model output prepared for CMIP6 ScenarioMIP ssp245 (Earth System Grid Federation). https://doi.org/10.22033/ESGF/CMIP6.8686.
8. John, J.G., Blanton, C., McHugh, C., Radhakrishnan, A., Rand, K., Vahlenkamp, H., Wilson, C., Zadeh, N.T., Dunne, J.P., et al. (2018). NOAA-GFDL GFDL-ESM4 model output prepared for CMIP6 ScenarioMIP ssp370 (Earth System Grid Federation). https://doi.org/10.22033/ESGF/CMIP6.8691.
9. Neubauer, D., Ferrachat, S., Siegenthaler-Le Drian, C., Stoll, J., Folini, D.S., Tegen, I., Wieners, K.-H.; Mauritsen, T., Stemmler, I., Barthel, S., et al. (2019). HAMMOZ-Consortium MPI-ESM1.2-HAM model output prepared for CMIP6 CMIP historical (Earth System Grid Federation). https://doi.org/10.22033/ESGF/CMIP6.5016.
10. Keeble, J., Hassler, B., Banerjee, A., Checa-Garcia, R., Chiodo, G., Davis, S., Eyring, V., Griffiths, P.T., Morgenstern, O., Nowack, P., et al. (2021). Evaluating stratospheric ozone and water vapour changes in CMIP6 models from 1850 to 2100. Atmospheric Chem. Phys. *21,* 5015-5061. https://doi.org/10.5194/acp-21-5015-2021.
11. Tang, Y., Rumbold, S., Ellis, R., Kelley, D., Mulcahy, J., Sellar, A., Walton, J., and Jones, C. (2019). MOHC UKESM1.0-LL model output prepared for CMIP6 CMIP historical (Earth System Grid Federation). https://doi.org/10.22033/ESGF/CMIP6.6113.
12. Good, P., Sellar, A., Tang, Y., Rumbold, S., Ellis, R., Kelley, D., and Kuhlbrodt, T. (2019). MOHC UKESM1.0-LL model output prepared for CMIP6 ScenarioMIP ssp126 (Earth System Grid Federation). https://doi.org/10.22033/ESGF/CMIP6.6339.
13. Good, P., Sellar, A., Tang, Y., Rumbold, S., Ellis, R., Kelley, D., and Kuhlbrodt, T. (2019). MOHC UKESM1.0-LL model output prepared for CMIP6 ScenarioMIP ssp245 (Earth System Grid Federation). https://doi.org/10.22033/ESGF/CMIP6.6339.
14. Good, P., Sellar, A., Tang, Y., Rumbold, S., Ellis, R., Kelley, D., and Kuhlbrodt, T. (2019). MOHC UKESM1.0-LL model output prepared for CMIP6 ScenarioMIP ssp370 (Earth System Grid Federation). https://doi.org/10.22033/ESGF/CMIP6.6347.
15. Good, P., Sellar, A., Tang, Y., Rumbold, S., Ellis, R., Kelley, D., and Kuhlbrodt, T. (2019). MOHC UKESM1.0-LL model output prepared for CMIP6 ScenarioMIP ssp585 (Earth System Grid Federation). https://doi.org/10.22033/ESGF/CMIP6.6339.
